# Autologous subcutaneous adipose tissue transplants improve adipose tissue metabolism and reduce insulin resistance and fatty liver in diet‐induced obesity rats

**DOI:** 10.14814/phy2.12909

**Published:** 2016-08-31

**Authors:** Gonzalo Torres‐Villalobos, Nashla Hamdan‐Pérez, Andrea Díaz‐Villaseñor, Armando R. Tovar, Ivan Torre‐Villalvazo, Guillermo Ordaz‐Nava, Sofía Morán‐Ramos, Lilia G. Noriega, Braulio Martínez‐Benítez, Alejandro López‐Garibay, Samuel Torres‐Landa, Juan C. Ceballos‐Cantú, Claudia Tovar‐Palacio, Elizabeth Figueroa‐Juárez, Marcia Hiriart, Roberto Medina‐Santillán, Carmen Castillo‐Hernández, Nimbe Torres

**Affiliations:** ^1^ Depto. de Cirugía Instituto Nacional de Ciencias Médicas y Nutrición Salvador Zubirán (INCMNSZ) Tlalpan Mexico City Mexico; ^2^ Depto. de Cirugía Experimental Instituto Nacional de Ciencias Médicas y Nutrición Salvador Zubirán (INCMNSZ) Tlalpan Mexico City Mexico; ^3^ Depto. de Fisiología de la Nutrición Instituto Nacional de Ciencias Médicas y Nutrición Salvador Zubirán (INCMNSZ) Tlalpan Mexico City Mexico; ^4^ Instituto de Investigaciones Biomédicas Universidad Nacional Autónoma de México Mexico City Mexico; ^5^ Depto. de Patología Instituto Nacional de Ciencias Médicas y Nutrición Salvador Zubirán (INCMNSZ) Tlalpan Mexico City Mexico; ^6^ Depto. de Nefrología y Metabolismo Mineral Instituto Nacional de Ciencias Médicas y Nutrición Salvador Zubirán (INCMNSZ) Tlalpan Mexico City Mexico; ^7^ Instituto de Fisiología Celular Universidad Nacional Autónoma de México Mexico City Mexico; ^8^ Departamento de Posgrado e Investigación Instituto Politécnico Nacional Escuela Superior de Medicina Mexico City Mexico

**Keywords:** Autologous adipose tissue transplant, diet‐induced obesity, insulin resistance, obesity

## Abstract

Long‐term dietary and pharmacological treatments for obesity have been questioned, particularly in individuals with severe obesity, so a new approach may involve adipose tissue transplants, particularly autologous transplants. Thus, the aim of this study was to evaluate the metabolic effects of autologous subcutaneous adipose tissue (SAT) transplants into two specific intraabdominal cavity sites (omental and retroperitoneal) after 90 days. The study was performed using two different diet‐induced obesity (DIO) rat models: one using a high‐fat diet (HFD) and the other using a high‐carbohydrate diet (HCHD). Autologous SAT transplant reduced hypertrophic adipocytes, improved insulin sensitivity, reduced hepatic lipid content, and fasting serum‐free fatty acids (FFAs) concentrations in the two DIO models. In addition, the reductions in FFAs and glycerol were accompanied by a greater reduction in lipolysis, assessed via the phosphorylation status of HSL, in the transplanted adipose tissue localized in the omentum compared with that localized in the retroperitoneal compartment. Therefore, the improvement in hepatic lipid content after autologous SAT transplant may be partially attributed to a reduction in lipolysis in the transplanted adipose tissue in the omentum due to the direct drainage of FFAs into the liver. The HCHD resulted in elevated fasting and postprandial serum insulin levels, which were dramatically reduced by the autologous SAT transplant. In conclusion, the specific intraabdominal localization of the autologous SAT transplant improved the carbohydrate and lipid metabolism of adipose tissue in obese rats and selectively corrected the metabolic parameters that are dependent on the type of diet used to generate the DIO model.

## Introduction

Obesity is an epidemic disease representing a particular focus of many public health efforts worldwide (Khan et al. 2009; Malik et al. 2013; Ogden et al. 2014). It is a complex metabolic disorder associated with the appearance of insulin resistance and dyslipidemia among other comorbidities; thus, there is an increased risk of developing type 2 diabetes and cardiovascular disease (Perrini et al. 2008).

The distribution of fat in obesity has a direct effect on metabolic abnormalities. It is known that there are several differences between subcutaneous adipose tissue (SAT) and visceral adipose tissue (VAT) (Ibrahim 2010). Some studies have demonstrated that obesity complications are mainly associated with an increase in the amount of VAT (Alvehus et al. 2010).

Different strategies have been used to prevent and treat obesity. However, the success of long‐term dietary and pharmacological treatments has been questioned, particularly in individuals with severe obesity (Kakkar and Dahiya 2015). For this reason, new strategies are being explored.

A new approach to the treatment of obesity may involve adipose tissue transplants. This is based on some animal studies that have demonstrated that heterologous and autologous adipose tissue transplants can improve several metabolic parameters, especially insulin sensitivity (Konrad et al. 2007; Tran et al. 2008; Foster et al. 2011, 2013; Satoor et al. 2011; Hocking et al. 2015). However, heterologous adipose tissue transplants in humans imply a risk of rejection, the use of immunosuppressants, and an increased risk of infection (Fishman 2007; Seetharam et al. 2010). Therefore, autologous adipose tissue transplant is a much more viable option that has been used for several years for esthetic purposes (Gutowski and Force 2009).

Adipose tissue transplants have been studied mostly in nonobese animals, and although some beneficial metabolic effects have been observed, these models do not represent the metabolic abnormalities that occur in obesity. To the best of our knowledge, few studies involving adipose tissue transplants have been conducted in diet‐induced obesity (DIO) models (Foster et al. 2013; Hocking et al. 2015). It has been reported that DIO models using a high‐fat diet (HFD) or a high‐carbohydrate diet (HCHD) induce different metabolic changes in humans (Thomas et al. 1992) and rodents (Buettner et al. 2007; Chaumontet et al. 2015; Torres‐Villalobos et al. 2015). However, whether an adipose tissue transplant can provide the same health benefits in obese animals depending on whether they developed obesity with a HFD or HCHD is unknown.

**Figure 1:**
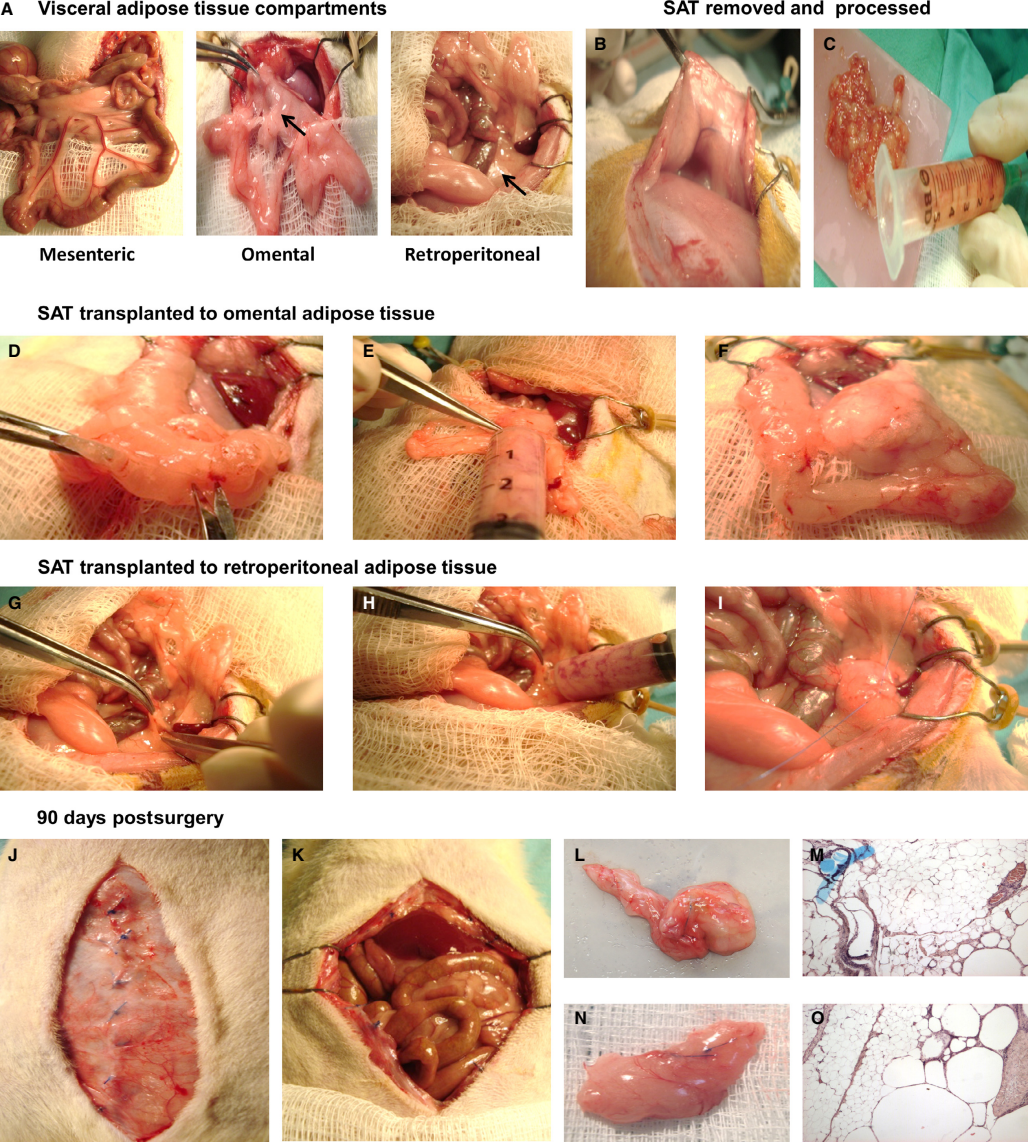
Visceral adipose tissue (VAT) compartments and transplant surgery process. (A) mesenteric, omental, and retroperitoneal VAT. Removal of subcutaneous adipose tissue (SAT) from both bilateral inguinal fat pads (B) and cut in small pieces that can pass through a syringe (C). Midventral abdominal incision was performed (D) and the SAT was injected between the two sheets of the omentum through the incision (E) and closed with nonabsorbable suture (F). Incision of the retroperitoneal adipose tissue (G) for the injection of the SAT (H), followed by the nonabsorbable suture (I). A duration of 90 days after the transplant, the site of the surgery did not present necrosis (J and K). The omental (L and M) and the retroperitoneal (N and O) adipose tissue were totally macro‐ and microscopically viable.

The inconsistencies between the reported metabolic effects of adipose tissue transplants are possibly related to differences in study duration and the use of obese or lean rodents (Konrad et al. 2007; Foster et al. 2011, 2013; Satoor et al. 2011; Hocking et al. 2015). In addition, it is important to consider the specific intraabdominal location where the adipose tissue is transplanted. Inside the abdominal cavity, three different fat compartments have been described, mesenteric, omental, and retroperitoneal adipose tissue (Fig. 1A). The venous flow of the intraperitoneal adipose tissue coming from the mesenteric and omental compartments is different than the venous flow of the retroperitoneum. The first two have their venous drainage to the portal vein into the liver, while the retroperitoneal compartment drains into the vena cava and directly to the systemic circulation (Tchernof and Despres 2013). This difference in venous drainage implies that all their products, such as free fatty acids (FFAs) and adipokines, will affect different targets and have been associated with different hypothesis regarding fat accumulation and disease. Therefore, it is important to consider the transplantation site because the metabolic products of the transplanted adipose tissue will reach different tissues (Rytka et al. 2011).

The mechanism by which the transplant improves metabolic parameters has not been well established. According to several studies, adipose tissue transplants can modify the flux of FFAs from adipose tissue to the liver (Foster et al. 2011, 2013; Hocking et al. 2015). It has been demonstrated that an increase in the drainage of FFAs from adipose tissue into the liver is associated with the development of insulin resistance (Ebbert and Jensen 2013). The release of FFAs from adipose tissue is dependent on the rate of lipolysis that is carried out by three different lipases. Specifically, the hormone‐sensitive lipase (HSL) and the adipose triglyceride lipase (ATGL) are the enzymes that highly regulate the lipolysis in adipocytes, however, obesity is associated with a decreased expression and activity of HSL, but not ATGL in visceral and subcutaneous adipocytes of obese individuals (Fruhbeck et al. 2014). Thus, HSL may be a key regulator of the flux of FFAs into the circulation after an adipose tissue transplant, although this has not been thoroughly studied.

Therefore, the aim of this study was to evaluate the metabolic effects of an autologous SAT transplant into two specific intraabdominal cavity sites (omental and retroperitoneal) using two different DIO rat models: one using a HFD and one using a HCHD. In addition, to establish whether the improvements in insulin sensitivity and hepatic lipid accumulation after adipose tissue transplant occur through modifications of adipose tissue lipolysis, the phosphorylation status of HSL in the omental and retroperitoneal adipose tissues was assessed.

## Materials and Methods

### Animals and diets

Male Wistar rats (8 weeks‐old) were housed in individual cages at controlled temperature (22°C) and humidity with a 12‐h light/dark cycle. The rats were fed a HFD or HCHD for 180 days. The HFD was composed of 18.7% of total energy from protein, 45% from fat, and 36.4% from carbohydrates. In addition, these rats consumed water supplemented with 5% sucrose. The HCHD was composed of 28.5% of total energy from protein, 13.5% from fat, and 57.9% from carbohydrates, as well as 20% sucrose in water; both groups were allowed free access to both food and water. The animal protocol was approved by the Animal Care and Research Advisory Committee of the Instituto Nacional de Ciencias Médicas y Nutrición Salvador Zubirán, Mexico City, Mexico (CEX‐35‐10‐11‐1).

### Experimental design and surgical procedures

**Figure 2:**
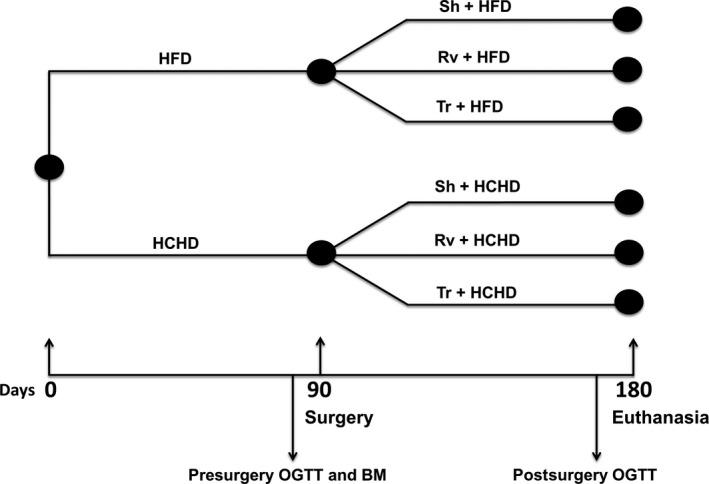
Experimental design. Rats were fed a high‐fat diet (HFD) or high‐carbohydrate diet (HCHD) for 180 days. The surgeries were achieved 90 days after the rats began to eat the experimental diets. After 90 extra days (180 days), euthanasia was performed to obtain liver and visceral adipose tissue (omental and retroperitoneal) samples. The oral glucose tolerance test (OGTT) with insulin measurements was performed a few days prior to surgery and euthanasia (presurgery and postsurgery). Blood samples from fasting rats for the analyses of biochemical parameters (BP) [glucose, insulin, and triglycerides (TGs)] were taken a few days prior to surgery (presurgery) and at euthanasia (postsurgery).

Once the rats developed obesity after consuming the HFD (*n* = 25) or HCHD (*n* = 14) for 90 days, they were randomly divided into three subgroups according to different surgical procedures: the sham group (Sh) (*n* = 8 for HFD and *n* = 5 for HCHD) which was the surgical control; the removal group (Rv) (*n* = 8 for HFD and *n* = 4 for HCHD); and the transplant group (Tr) (*n* = 9 for HFD and *n* = 5 for HCHD) (Fig. 2).

For surgery, inhalation of 2% sevoflurane with oxygen at 0.4–0.6 L/min was used to establish anesthesia, and on demand, sevoflurane with oxygen at 0.4–0.6 L/min was used for maintenance, provided by a Kent Scientific ventilator. The Sh group underwent laparotomy and remained under anesthesia for the same amount of time as the other two groups. For the Rv group, SAT was completely removed from both bilateral inguinal fat pads without being transplanted (Fig. 1B). For the Tr group, the SAT that was extracted from the same area in the inguinal fat pad, was weighed and cut in small pieces that could pass through a syringe (Fig. 1B and C) to implant them into two specific intraabdominal compartments: omental and retroperitoneal. A midventral abdominal incision was performed and the SAT (3.15 ± 1.1 g) was injected between the two sheets of the omentum through a small incision and closed with nonabsorbable suture to recognize the transplant site (Table 1 and Fig. 1D–F). Then, the retroperitoneal adipose tissue was localized in both sides, and the SAT was injected (5.8 ± 2.2 g) through an opening in the retroperitoneum. The incision was closed in the same manner as in the omentum (Fig. 1G–I). After the surgery, all animals received prophylactic analgesia (meloxicam 0.4 mg/kg) and an intramuscular antibiotic (enrofloxacin) for 2 and 3 days, respectively.

**Table 1:** Body and adipose tissue weight

	HFD			HCHD		
	Sham	Removal	Transplant	Sham	Removal	Transplant
Body weight (g)						
Initial	275.70 ± 20	285.95 ± 26	259.42 ± 32	218.13 ± 22	246.47 ± 20	232.69 ± 14
Presurg	589.6 ± 11.49	589.3 ± 17.05	582.2 ± 15.8	601.9 ± 28.5	567.5 ± 24.28	593.9 ± 12.8
Postsurg	673 ± 3.7	639.2 ± 6.3*	630.8 ± 2.5*	717.9 ± 5	571.8 ± 8.4*	601.2 ± 1.7*
SAT transplanted to (g)						
Omental	N/A	N/A	3 ± 0.48	N/A	N/A	3.5 ± 0.28
Retroperitoneal	N/A	N/A	5.85 ± 0.95	N/A	N/A	5.66 ± 0.72
Adipose tissue weight (g)						
Subcutaneous	23.33 ± 3.2	12.87 ± 3.2**	12.61 ± 2.3**	38.84 ± 2.1	6.8 ± 2.5*	22.67 ± 2.6*
Visceral	55.3 ± 2.1	55.4 ± 4.0	54.71 ± 2.0	58.22 ± 3.2	37.08 ± 3.1*	22.67 ± 3.4*
Energy consumption (kcal)	144.1 ± 4.4	132.5 ± 8.1	140.8 ± 4.0	165.9 ± 12.3	142.1 ± 14.4	168.2 ± 18.4
Liver weight (g)	17.08 ± 1.1	14.53 ± 1.2	14.10 ± 1.0	15.67 ± 1.3	15.59 ± 1.0	14.21 ± 0.6

The values are the mean ± SEM. The symbols denote significant differences versus sham *P < 0.05 and **P < 0.001. Not applicable (N/A).

Afterward, the rats continued the same experimental diet (HFD or HCHD) that was assigned at the beginning of the study for 90 days after surgery, completing a total of 180 days (Fig. 2). As shown in Figure 1J–O, 90 days after the surgery there was no necrosis on the surgery wound (Fig. 1J and K) or in the two transplanted tissues, that were macro and microscopically completely viable. Moreover, through the blue nonabsorbable suture, it can be distinguished also that the transplant is still present after 90 days of the surgery (Fig. 1L–O).

Body weight was monitored every 3 days. One week before surgery, fasting glucose, insulin, and triglycerides (TGs) were measured, and an oral glucose tolerance test (OGTT) was performed, with simultaneous insulin measurements. One week before euthanasia, the OGTT was repeated, with simultaneous insulin measurements (Fig. 2). Euthanasia was performed by decapitation after CO_2_ anesthetization. Blood was collected in tubes containing a separating gel and clot activator (BD Franklin Lakes, NJ) and centrifuged at 1500 × g for 10 min to measure fasting serum glucose, insulin, TGs, glycerol, and FFAs. Liver and VAT (omental and retroperitoneal) samples were dissected, weighed, and maintained at −70°C until analysis, whereas SAT was only dissected and weighed. Liver and VAT samples were also fixed for histological assessment.

### Oral glucose tolerance test

An Oral glucose tolerance test (OGTT) was performed one week before surgery (presurg) or before euthanasia (postsurg) (Fig. 2). Briefly, the rats were fasted overnight (12 h), and blood samples were collected from the tail vein before and 15, 30, 60, 90, 120, and 150 min after administration of the glucose load. Glucose was administered intragastrically at a dose of 2 g/kg of body weight with a 30% glucose solution. Serum glucose and serum insulin concentrations were measured at each point.

### Determination of serum biochemical parameters

Serum glucose was quantified by the glucose oxidase method using a YSI 2700 Select Biochemistry Analyzer (YSI Incorporated, Yellow Spring, OH). The concentration of insulin was measured by radioimmunoassay (RIA) (Linco Research Immunoassay, Millipore) according to the manufacturer's instructions. Serum TGs (DiaSys Diagnostic System, Holzheim Germany), FFAs (Roche Diagnostics GmbH & Roche Applied Science, Nonnenwald, Germany), and glycerol (Sigma‐Aldrich, St. Louis, MO), were determined using enzymatic‐colorimetric assay kits. All of the above parameters in each sample were analyzed in duplicate. Fasting insulin resistance was evaluated indirectly using the insulin resistance (IR) index and was calculated as follows: [fasting glucose (mmol/L) × fasting insulin (μU/mL)]/22.5.

### Hepatic content of triglycerides and cholesterol

Hepatic lipids were extracted using the Folch method (Folch et al. 1957), and TG and cholesterol content were quantified by enzymatic‐colorimetric kits (DiaSys Diagnostic System, Holzheim Germany).

### Histological analysis

Liver and VAT (omental and retroperitoneal) samples were fixed in an ice‐cold 4% (w/v) formaldehyde in phosphate‐buffered saline (PBS) and were embedded in paraffin. Then, sections of 4 μm were stained with hematoxylin–eosin. Images were obtained at 40× and 10× magnification, for liver and adipose tissue samples, respectively.

### Western blotting

Proteins were extracted from omental and retroperitoneal adipose tissue and were quantified as described previously (Diaz‐Villasenor et al. 2013). Then, 10 μg of total protein was denatured by heating for 5 min in a Laemmli sample buffer containing *β*‐mercaptoethanol (Bio‐Rad, Hercules, CA), separated by SDS‐PAGE using 8% polyacrylamide gels, and transferred to PVDF membranes. The blotted membranes were blocked for 1 h at room temperature using 5% nonfat dry milk (Bio‐Rad, Hercules CA) and were then incubated overnight at 4°C with the following primary antibodies: HSL #45422 diluted 1:140,000 or UCP‐1 #10983 diluted 1:2000 from Abcam (Cambridge, MA), pHSLSer563 #4139 diluted 1:750 from Cell Signaling Technology (Danvers, MA), and Actin #1615 diluted 1:1500 from Santa Cruz Biotechnology (Santa Cruz, CA). After incubation with the secondary antibody, the blots were developed using the enhanced chemiluminescence method with Immobilon Western Chemiluminescent HRP substrate (Millipore, Billerica, MA). The labeled bands were visualized using the ChemiDoc MP Imaging System (BioRad, Hercules CA). Optical densitometric analysis was conducted using ImageJ 1.42p digital imaging processing software (Rasband 1997). The immunoblotting assays were performed with three independent blots, and the values were normalized relative to actin values.

### Statistical analysis

The values are expressed as the mean ± SEM. The pre‐ and postsurgical differences were analyzed by two‐way ANOVA test followed by Bonferroni′s multiple comparison test. Differences between the AUCs of presurgery rats fed the HFD or HCHD were evaluated with one‐sided unpaired *t*‐tests (using Welch′s correction when the variance between the groups was significantly different) or Mann–Whitney tests (for nonparametric data), as indicated in each figure. Differences between the experimental groups (Rv and Tr) and the control group (Sh) were evaluated using one‐way ANOVA followed by Dunnett's multiple comparison test or the Kruskal–Wallis test followed by Dunn's multiple comparison test for nonparametric data, as indicated in each figure. The normality of the data distribution was evaluated using the Kolmogorov–Smirnov test. In all cases, *P* < 0.05 was considered significant (GraphPad Prism 5.00, San Diego, CA).

## Results

### Diet‐induced obesity and presurgical metabolic changes after the consumption of a HFD or HCHD

The appearance of metabolic abnormalities differs when obesity is induced using a HFD compared with a HCHD (Thomas et al. 1992; Buettner et al. 2007; Chaumontet et al. 2015; Torres‐Villalobos et al. 2015). Therefore, to assess the impact of an autologous fat transplant from the subcutaneous fat pad to the visceral compartments, we developed a diet‐induced obesity (DIO) using a HFD or HCHD. We observed that rats fed a HFD gained approximately 313 g after 90 days of dietary treatment, whereas those fed a HCHD gained approximately 355 g during the same period (Table 1).

**Figure 3:**
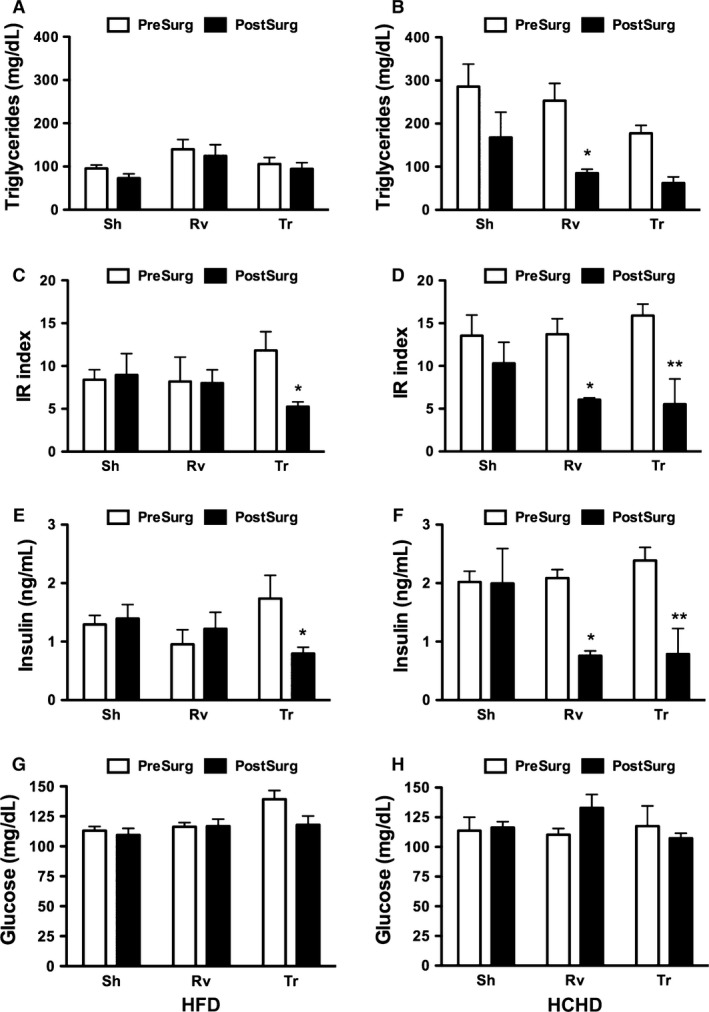
Biochemical parameters before and after surgery. Fasting serum triglycerides (TGs) (A and B), insulin resistance (IR) index (C and D), fasting serum insulin (E and F), and fasting serum glucose, (G and H) were determined in rats fed the high‐fat diet (HFD) (A, C, E, and G) and in rats fed the high‐carbohydrate diet (HCHD) (B, D, F, and H) a few days prior to surgery (presurgery) and 90 days after surgery (postsurgery) in the sham (Sh), removal (Rv), and transplant (Tr) groups. The data are presented as the mean ± SEM and differences were significant at *P < 0.05 and **P < 0.01 between the rats presurgery and postsurgery evaluated by two‐way ANOVA.

**Figure 4:**
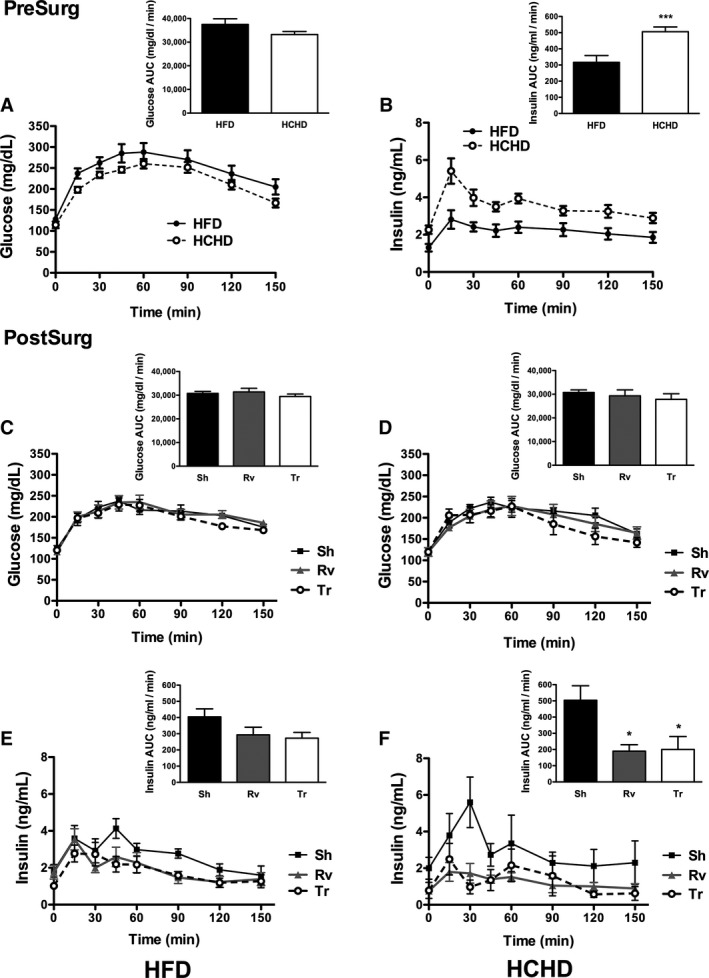
Oral glucose tolerance test (OGTT) with insulin measurements. A few days prior to surgery (presurgery), an OGTT (A) with insulin measurements (B) was performed in rats fed a HFD and a HCHD. A duration of 90 days after surgery and a few days prior to euthanasia (postsurgery), an OGGT was performed in rats fed the HFD (C) and the HCHD (E), with simultaneous insulin measurements (D and F) in the sham (Sh), removal (Rv), and transplant (Tr) groups. In the right upper corner of each figure, the AUC is shown. The data are presented as the mean ± SEM, and differences were significant at ***P < 0.001 between the rats fed the HFD and HCHD presurgery and *P < 0.05 compared with the Sh condition postsurgery. Statistical differences in the AUC presurgery were evaluated using the Mann–Whitney test for glucose and a one‐sided unpaired *t*‐test for insulin, and the AUC in the postsurgery group was assessed using one‐way ANOVA followed by the Dunnett analysis for glucose with both diets and for insulin in rats fed the HCHD and the Kruskal–Wallis test followed by the Dunn posttest for insulin in rats fed the HFD.

As a result of 90 days of dietary treatment with both diets and despite the lack of a significant difference in weight gain between the groups, rats fed a HCHD had a significantly higher fasting serum TGs (Fig. 3A and B) and IR index (Fig. 3C and D) than those fed a HFD. The IR index and fasting serum TGs were 34% (*P* = 0.03) and 102% (*P* = 0.0001) greater, respectively, in the group fed the HCHD than the group fed the HFD (Fig. 3A–D). Nonetheless, in both groups, the IR index and fasting serum TGs were above the previously reported control values (Eu et al. 2010; Kowalski and Bruce 2014). We observed that the elevation in the IR index was the result of an increase in serum insulin without a significant change in fasting serum glucose in both groups (Fig. 3E–H). In addition, this difference between rats fed a HCHD and rats fed a HFD was evident after the OGTT with insulin measurements because the area under the curve (AUC) for serum insulin was significantly greater in rats fed a HCHD than in rats fed a HFD (Fig. 4A and B).

### Body weight change after autologous transplant in obese rats fed a HFD or HCHD

A duration of 90 days after the surgical procedure (Fig. 2), the Sh group fed a HFD gained 83 g, whereas the corresponding Rv and Tr groups gained approximately 49.9 g and 48.6 g, respectively, representing 40.1% and 41.7% significantly less weight gain than the Sh group (Table 1). Interestingly, the Sh group fed a HCHD gained 116 g, but surprisingly, the Rv and Tr groups gained approximately 4.3 and 7.3 g, respectively, representing 96.2% and 93.7% significantly less weight gain than the Sh group. However, energy consumption and liver weight were similar among the three groups for each diet (Table 1).

At the end of the study, as expected, the amount of SAT in rats fed a HFD was significantly less in the Rv and Tr groups than in the Sh group because it was removed during the surgical procedure (Table 1). However, despite the amount of SAT transplanted to VAT in the Tr group, there was not a significant difference among the groups in the amount of visceral fat (Table 1). Regarding the groups of rats fed a HCHD, the Rv and Tr groups also had significantly less SAT than the Sh group. Nonetheless, there was less subcutaneous fat in the Rv group than in the Tr group. In contrast to the rats fed a HFD, the rats fed a HCHD showed significantly less VAT in the Rv and Tr groups than in the Sh group (Table 1). The ratio of visceral to subcutaneous fat was 5.4 in the Rv group, whereas in the Tr group, this ratio was 1.0. Interestingly, in the Tr group, the amount of VAT was even less than in the Rv group, although the SAT was transplanted to the visceral compartment (Table 1).

### Metabolic changes after autologous transplant in obese rats

There was a marked difference in some metabolic parameters in rats fed a HFD or HCHD. In rats fed either a HFD or HCHD, there was a trend to reduce fasting serum glucose; however, this difference did not reach statistical significance (Fig. 3G and H). Nonetheless, fasting serum insulin was significantly reduced after the transplant with both diets. Rats fed a HFD and HCHD had 54% and 67% less insulin, respectively, than prior to surgery (Fig. 3E and F). As a consequence, there was a significant reduction in the IR index of 59% and 65% in the Tr groups fed a HFD and HCHD, respectively (Fig. 3C and D). Presurgical fasting serum TG concentrations in rats fed a HCHD were greater than those in rats fed a HFD. In rats fed a HFD, fasting serum TG values were similar before and after surgery. However, in rats fed a HCHD, fasting serum TGs tended to reduce after transplant (Fig. 3A and B). Unexpectedly, the Rv group fed a HCHD showed a reduction in fasting serum insulin, IR index, and fasting serum TGs (Fig. 3B, D, and F). This was an effect not observed in the same group fed the HFD (Fig. 3A, C, and E).

### Autologous transplant in obese rats improves insulin sensitivity

To further determine changes in insulin sensitivity, we performed an OGTT with insulin measurements 90 days after surgery (Fig. 4C–F). The AUC values of serum glucose of the OGTT were not modified among the three groups in both diets (Fig. 4C and D). The AUC of the serum insulin concentrations of the rats fed a HFD tended to decrease in the Tr group compared with the Sh group (Fig. 4E). Nevertheless, in rats fed a HCHD, the AUC was significantly reduced in the Tr group compared to the Sh group (Fig. 4F), indicating that less insulin was required to maintain the same serum glucose concentrations.

### Hepatic lipid reduction after autologous transplant in obese rats

**Figure 5:**
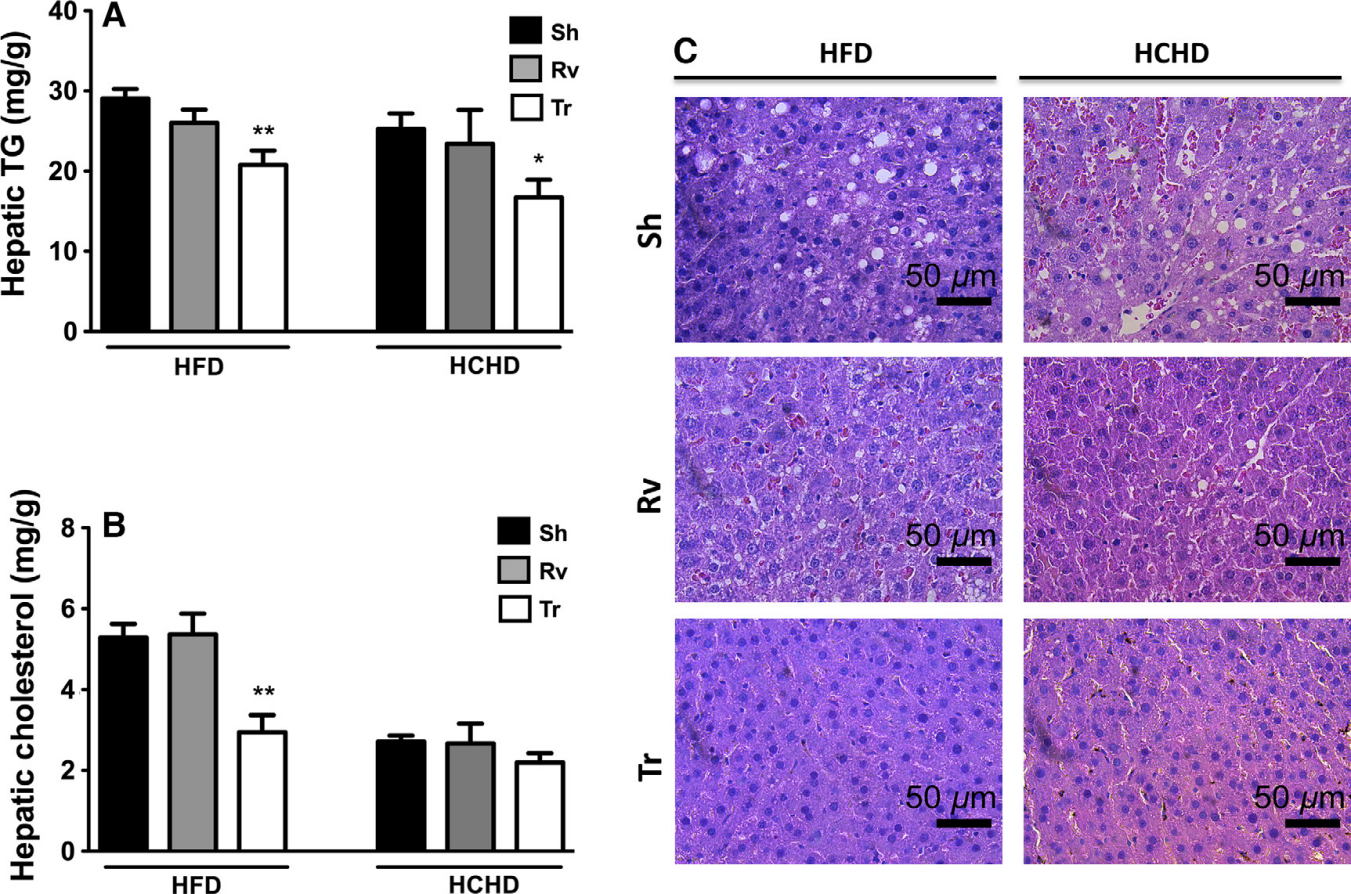
Hepatic lipid content. TG (A) and cholesterol (B) content was determined in the liver of rats fed the HFD or the HCHD 90 days after surgery in the sham (Sh), removal (Rv), and transplant (Tr) rats. Data are presented as the mean ± SEM, and differences were significant at *P < 0.05 and **P < 0.01 compared with the Sh condition. Differences in the content of hepatic TGs in rats fed both diets and in the cholesterol content in rats fed the HCHD were evaluated using the Kruskal–Wallis test followed by the Dunn test, whereas liver cholesterol content in rats fed the HFD was assessed using ANOVA followed by the Dunnett test. Representative images of liver samples stained with eosin and hematoxylin from rats fed the HFD or the HCHD 90 days after surgery in the sham (Sh), removal (Rv), and transplant (Tr) groups (C).

Hepatic TG concentrations were significantly reduced only in the Tr group of rats fed a HFD or HCHD (Fig. 5A). Rats fed a HFD showed significantly lower hepatic cholesterol concentrations in the Tr group than in the Sh or Rv groups. However, rats fed a HCHD maintained a normal hepatic cholesterol concentration that did not change with the transplant (Fig. 5B). These findings correlated with the histological analysis that revealed that the liver showed fewer lipid deposits only in the case of the autologous transplant (Fig. 5C).

### Autologous transplant reduced FFAs through a decrease in adipose tissue lipolysis

**Figure 6:**
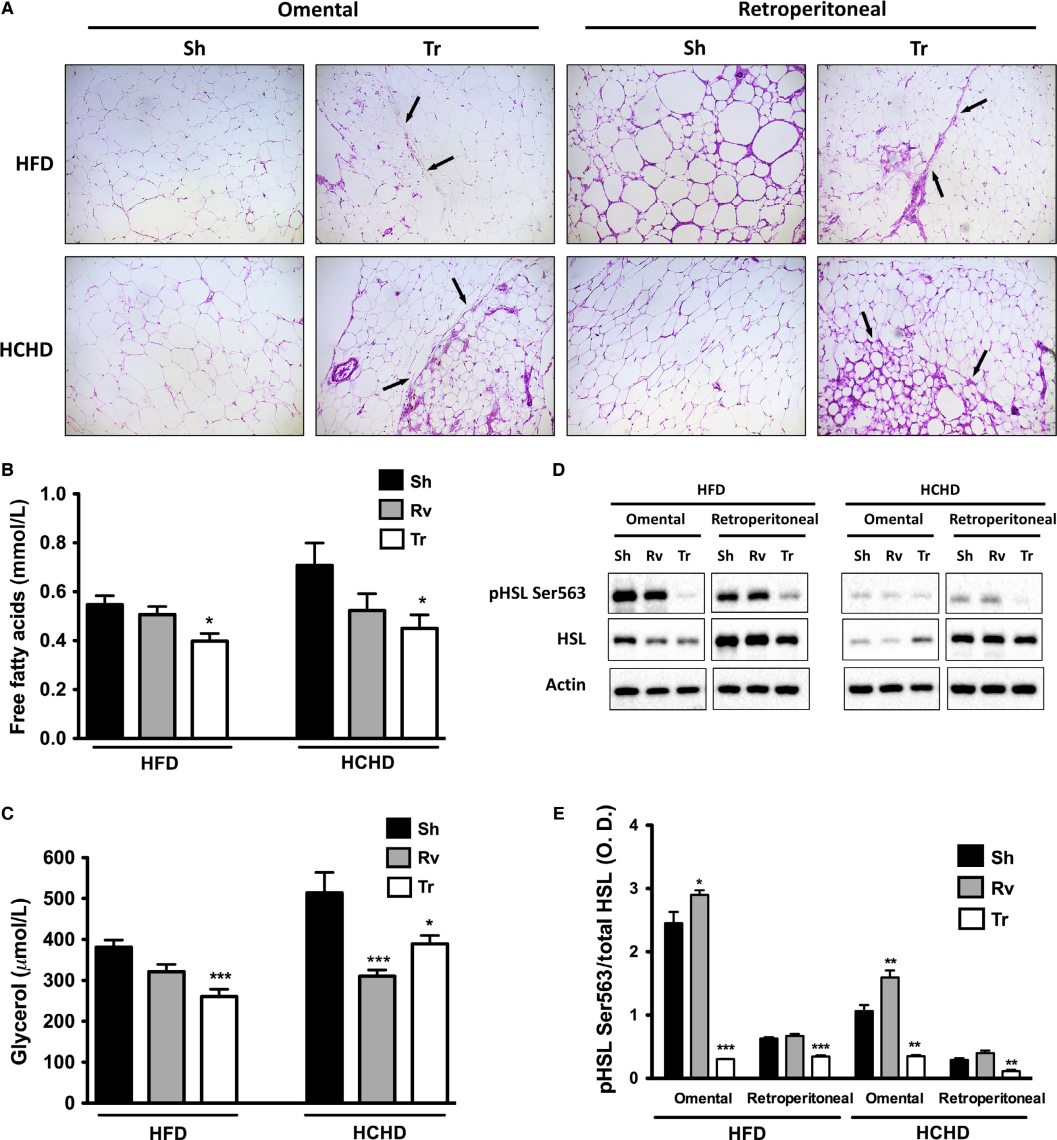
Adipose tissue morphology and lipolytic functionality. Representative images of the omental and retroperitoneal adipose tissue samples stained with hematoxylin and eosin from rats fed the HFD or the HCHD 90 days after surgery in the sham (Sh) and transplant (Tr) groups (A). Fasting serum FFAs (B) and glycerol concentrations (C) in rats fed the HFD and the HCHD 90 days after surgery in the sham (Sh), removal (Rv), and transplant (Tr) groups. Representative blots of pHSL Ser563, total HSL, and actin in the omental and retroperitoneal adipose tissue of rats fed the HFD and the HCHD 90 days after surgery in the sham (Sh), removal (Rv), and transplant (Tr) groups (D), and the ratio of pHSL Ser563 to HSL was evaluated via optical density analyses of the blots normalized to actin (E). The data are presented as the mean ± SEM, and symbols denote significant differences between the removal (Rv) or transplant (Tr) groups versus the Sham group (Sh) at *P < 0.05, **P < 0.01 and ***P < 0.001. Significant differences for free fatty acids (FFAs) in rats fed the HFD and glycerol in both diets were evaluated using the Kruskal–Wallis test followed by the Dunn posttest, whereas FFAs in the rats fed the HCHD and the ratio of the phosphorylation of HSL were determined by ANOVA followed by the Dunnett posttest.

In the histology of the omental and retroperitoneal adipose tissues, two cell populations of different size can be distinguished in the Tr group (indicated by arrows), in which the smaller adipocytes corresponded to the transplanted SAT, whereas in the Sh group, only hypertrophic adipocytes were observed. This was more evident in rats fed HCHD than HFD (Fig. 6A).

The mitochondrial uncoupling protein 1 (UCP‐1) is present in brown and beige adipocytes, whereas white adipocytes are UCP‐1‐negative. The function of UCP‐1 is to uncouple electron transport from ATP production, which in turn leads to controlled exothermic resolution of the electrochemical gradient and generation of heat (Harms and Seale 2013; Bartelt and Heeren 2014). Thus, since the rats that were transplanted with SAT had less or the same weight of VAT as the Sh or Rv groups (Table 1), the expression of UCP‐1 was evaluated in the omental and retroperitoneal adipose tissue as a browning marker. However, UCP‐1 levels did not increase in the Tr group in comparison with the Sh and Rv groups (data not shown).

There is evidence that an increase in circulating FFAs causes hepatic and muscle lipid accumulation, leading to insulin resistance and lipotoxicity (Fruhbeck et al. 2014). After the transplant, fasting serum FFAs were significantly reduced compared with the Sh group in both diets (Fig. 6B). Fasting serum glycerol, which was utilized as a lipolysis marker, was also significantly reduced in the Tr group compared with the Sh group (Fig. 6C). To evaluate the mechanism by which lipolysis was decreased with the autologous transplant, the activity of hormone‐sensitive lipase (HSL) was measured through its phosphorylation at Ser563 in the omental or retroperitoneal adipose tissue where the SAT was transplanted.

The abundance of total HSL protein, as well as pHSL at Ser563, was greater in rats fed a HFD than in rats fed a HCHD in both compartments (omental and retroperitoneal) (Fig. 6D). The proportion of pHSL at Ser563 in relation to total HSL content was significantly reduced in the Tr group compared with the Sh and Rv groups with both diets in both compartments. Finally, this effect was much more evident in the adipose tissue transplanted to the omental compartment than the adipose tissue transplanted to the retroperitoneal compartment in both diets (Fig. 6D and E).

## Discussion

Autologous SAT transplant improved the metabolic parameters associated with the DIO model. A duration of 90 days after SAT transplant into the omental and retroperitoneal intraabdominal compartments in the HFD‐ or HCHD‐induced obesity models, insulin sensitivity was improved, hepatic lipid content was reduced, and FFAs concentrations were also decreased.

It is interesting to remark on the significant reduction in the amount of VAT in the Tr group compared with the Sh group in rats fed a HCHD, taking into account that in the Tr group, the SAT was removed and then transplanted into the VAT. However, this effect was not observed in rats fed a HFD, suggesting that energy substrates play an important role in this finding. In fact, we have previously reported that adipose tissue metabolism depends on diet composition (Frigolet et al. 2011; Tovar et al. 2011; Diaz‐Villasenor et al. 2013). Therefore, this reduction in VAT after the transplant may explain the significant improvement in insulin sensitivity, and consequently, the remarkable reduction in body weight in rats fed the HCHD. This reduction in the mass of the VAT and the metabolic improvement observed with the transplant was not associated with WAT browning, nevertheless is probably associated with the shift in the size of the adipocytes, since small adipocytes are more insulin sensitive and functional than hypertrophic adipocytes (Laforest et al. 2015). However, future studies will be required to understand the mechanism by which VAT was decreased in rats fed a HCHD after the transplant.

The mechanism by which the transplant improves metabolic parameters is still unknown. Although there is no consensus regarding the site where the transplant should be located, we believe that not only the type of adipose tissue that is transplanted (SAT or VAT) but also the intraabdominal localization of the transplant plays a major role in the metabolic effects.

It has been reported in several articles that serum FFAs are reduced after adipose tissue transplant (Gavrilova et al. 2000; Foster et al. 2011, 2013; Hocking et al. 2015). However, approximately one‐third of the FFAs released from the adipose tissue to the circulation are reesterified, so a more accurate marker of lipolysis is the measurement of serum glycerol (Forest et al. 2003). Here, we demonstrate that both serum FFAs and glycerol decreased after the transplant in both DIO models, indicating that less lipolysis occurs in these rats, which possibly explains the observed reduction in hepatic lipid content. In fact, the venous drainage of the omentum goes directly to the portal system that ultimately reaches the liver, whereas the venous drainage of the retroperitoneum goes to the systemic circulation through the vena cava (Rytka et al. 2011; Tchernof and Despres 2013).

An important step in lipolysis activation in response to catecholaminergic stimulation comprises the translocation of HSL from a cytosolic compartment to the surface of the lipid droplet, through the protein kinase A‐mediated phosphorylation of HSL at Ser563, Ser659, and Ser660 (Fruhbeck et al. 2014). Accordingly, the decrease in the active state of HSL, evaluated through the phosphorylation at Ser563, was significantly more evident in the omental compartment than in the retroperitoneal compartment. Therefore, the improvement in hepatic lipid content after autologous SAT transplant may be attributed to a reduction in lipolysis from the transplanted adipose tissue localized in the omentum, also in accordance to the size of the adipocytes.

In addition, the removal of SAT in rats fed the HCHD and HFD had different outcomes. To understand the metabolic changes observed after the removal of SAT from obese rats fed a HCHD, it is necessary to assess the metabolic changes induced by the HCHD, which occurred in the Sh group. In rats subjected to the sham procedure, consumption of the HCHD significantly increased fasting and circulating postprandial insulin levels, leading to an increase in the amount of SAT, possibly due to an increase in lipogenesis (Wong and Sul 2010). As a consequence, there was an increase in the fasting plasma concentrations of FFAs and glycerol, suggesting increased lipolysis from SAT associated with concomitant insulin resistance (Rosen and Spiegelman 2006; Patel and Abate 2013). In fact, these rats had an increased IR index, which likely contributed to a feedback loop mechanism that increases the hyperinsulinemia, which has been previously reported (Kahn 2003). Furthermore, the elevated levels of insulin can also stimulate hepatic lipogenesis, contributing to the synthesis of VLDL particles, which leads to an elevation in circulating TGs and can increase the accumulation of skeletal muscle TGs, exacerbating the insulin resistance (Wong and Sul 2010). Therefore, the export capacity of the liver to transfer TGs into the circulation via VLDL prevents accumulation of lipid depots in this organ (Ebbert and Jensen 2013). With SAT removal, most of the metabolic abnormalities induced in the obese rats fed the HCHD were ameliorated in part due to decreased adipose tissue fat lipolysis, resulting in reduced circulating glycerol levels and improved insulin sensitivity.

Conversely, rats fed the HFD had an elevated flux of TGs into the liver from the diet (Torres‐Villalobos et al. 2015). Sh obese rats had an increase in VAT lipolysis associated with an increment in the active form of HSL. These rats also had high circulating insulin levels; however, these levels were approximately 40% lower than in the rats fed the HCHD. It is possible that this result was mainly a consequence of an accumulation of lipids in the liver without an increase in the amount of SAT. We believe that this resulted in less lipolysis from adipose tissue, attenuating the accumulation of lipids in skeletal muscle, leading to a decrease in insulin resistance, as demonstrated by the lower IR index compared with that in obese rats fed the HCHD. Interestingly, the removal of SAT in obese rats fed the HFD did not have a beneficial metabolic effect.

In rats fed either the HCHD or the HFD, the transplant of SAT generated metabolic improvements in both groups. The mechanism that we proposed is that in the omental and retroperitoneal adipose tissue transplanted with SAT, the size of the adipocytes became less hypertrophic, thus improving adipose tissue insulin sensitivity, resulting in greater insulin‐mediated suppression of lipolysis, since insulin antagonizes catecholamine‐induced lipolysis. Thus, fasting lipolysis was reduced due to a decrease in the active state of HSL in VAT, and in consequence, there was a decrease in circulating FFAs and glycerol. A decreased flux of FFAs to circulation impacted on less lipid accumulation in liver and probably in muscle, consequently improving systemic insulin sensitivity evaluated through the IR index and resulting in lower serum insulin levels. As a consequence, several metabolic variables were improved compared with the Sh obese rats. However, more studies are needed to understand the molecular mechanism by which the transplant can modulate the activation of HSL.

In conclusion, our study clearly showed that an autologous SAT transplant may be considered an important strategy to improve the homeostasis of carbohydrate and lipid metabolism during obesity. It is necessary to take into account that the location of the SAT transplant into the visceral compartments of adipose tissue is an important issue to generate beneficial health effects. Further studies are needed to demonstrate that the use of autologous SAT transplant can be utilized in humans with obesity.

## Conflict of Interest

None declared.

## References

[phy212909-bib-0001] Alvehus, M. , J. Buren , M. Sjostrom , J. Goedecke , and T. Olsson . 2010 The human visceral fat depot has a unique inflammatory profile. Obesity 18:879–883. 20186138 10.1038/oby.2010.22

[phy212909-bib-0002] Bartelt, A. , and J. Heeren . 2014 Adipose tissue browning and metabolic health. Nat. Rev. Endocrinol. 10:24–36. 24146030 10.1038/nrendo.2013.204

[phy212909-bib-0003] Buettner, R. , J. Scholmerich , and L. C. Bollheimer . 2007 High‐fat diets: modeling the metabolic disorders of human obesity in rodents. Obesity (Silver Spring) 15:798–808. 17426312 10.1038/oby.2007.608

[phy212909-bib-0004] Chaumontet, C. , D. Azzout‐Marniche , A. Blais , T. Chalvon‐Dermersay , N. A. Nadkarni , J. Piedcoq , et al. 2015 Rats prone to obesity under a high‐carbohydrate diet have increased post‐meal CCK mRNA expression and characteristics of rats fed a high‐glycemic index diet. Front. Nutr. 2:22. 26217667 10.3389/fnut.2015.00022 PMC4497311

[phy212909-bib-0005] Diaz‐Villasenor, A. , O. Granados , B. Gonzalez‐Palacios , C. Tovar‐Palacio , I. Torre‐Villalvazo , V. Olivares‐Garcia , et al. 2013 Differential modulation of the functionality of white adipose tissue of obese Zucker (fa/fa) rats by the type of protein and the amount and type of fat. J. Nutr. Biochem. 24:1798–1809. 23773624 10.1016/j.jnutbio.2013.03.007

[phy212909-bib-0006] Ebbert, J. O. , and M. D. Jensen . 2013 Fat depots, free fatty acids, and dyslipidemia. Nutrients 5:498–508. 23434905 10.3390/nu5020498 PMC3635208

[phy212909-bib-0007] Eu, C. H. , W. Y. Lim , S. H. Ton , and K. Bin Abdul Kadir . 2010 Glycyrrhizic acid improved lipoprotein lipase expression, insulin sensitivity, serum lipid and lipid deposition in high‐fat diet‐induced obese rats. Lipids Health Dis. 9:81. 20670429 10.1186/1476-511X-9-81 PMC2927592

[phy212909-bib-0008] Fishman, J. A. 2007 Infection in solid‐organ transplant recipients. N. Engl. J. Med. 357:2601–2614. 18094380 10.1056/NEJMra064928

[phy212909-bib-0009] Folch, J. , M. Lees , and G. H. Sloane Stanley . 1957 A simple method for the isolation and purification of total lipides from animal tissues. J. Biol. Chem. 226:497–509. 13428781

[phy212909-bib-0010] Forest, C. , J. Tordjman , M. Glorian , E. Duplus , G. Chauvet , J. Quette , et al. 2003 Fatty acid recycling in adipocytes: a role for glyceroneogenesis and phosphoenolpyruvate carboxykinase. Biochem. Soc. Trans. 31:1125–1129. 14641009 10.1042/bst0311125

[phy212909-bib-0011] Foster, M. T. , H. Shi , S. Softic , R. Kohli , R. J. Seeley , and S. C. Woods . 2011 Transplantation of non‐visceral fat to the visceral cavity improves glucose tolerance in mice: investigation of hepatic lipids and insulin sensitivity. Diabetologia 54:2890–2899. 21805228 10.1007/s00125-011-2259-5 PMC5451325

[phy212909-bib-0012] Foster, M. T. , S. Softic , J. Caldwell , R. Kohli , A. D. De Kloet , and R. J. Seeley . 2013 Subcutaneous adipose tissue transplantation in diet‐induced obese mice attenuates metabolic dysregulation while removal exacerbates it. Physiol. Rep. 1:e00015. 23914298 10.1002/phy2.15 PMC3728904

[phy212909-bib-0013] Frigolet, M. E. , N. Torres , L. Uribe‐Figueroa , C. Rangel , G. Jimenez‐Sanchez , and A. R. Tovar . 2011 White adipose tissue genome wide‐expression profiling and adipocyte metabolic functions after soy protein consumption in rats. J. Nutr. Biochem. 22:118–129. 20471815 10.1016/j.jnutbio.2009.12.006

[phy212909-bib-0014] Fruhbeck, G. , L. Mendez‐Gimenez , J. A. Fernandez‐Formoso , S. Fernandez , and A. Rodriguez . 2014 Regulation of adipocyte lipolysis. Nutr. Res. Rev. 27:63–93. 24872083 10.1017/S095442241400002X

[phy212909-bib-0015] Gavrilova, O. , B. Marcus‐Samuels , D. Graham , J. K. Kim , G. I. Shulman , A. L. Castle , et al. 2000 Surgical implantation of adipose tissue reverses diabetes in lipoatrophic mice. J. Clin. Invest. 105:271–278. 10675352 10.1172/JCI7901 PMC377444

[phy212909-bib-0016] Gutowski, K. A. , and A. F. G. T. Force . 2009 Current applications and safety of autologous fat grafts: a report of the ASPS fat graft task force. Plast. Reconstr. Surg. 124:272–280. 19346997 10.1097/PRS.0b013e3181a09506

[phy212909-bib-0017] Harms, M. , and P. Seale . 2013 Brown and beige fat: development, function and therapeutic potential. Nat. Med. 19:1252–1263. 24100998 10.1038/nm.3361

[phy212909-bib-0018] Hocking, S. L. , R. L. Stewart , A. E. Brandon , E. Suryana , E. Stuart , E. M. Baldwin , et al. 2015 Subcutaneous fat transplantation alleviates diet‐induced glucose intolerance and inflammation in mice. Diabetologia 58:1587–1600. 25899451 10.1007/s00125-015-3583-y

[phy212909-bib-0019] Ibrahim, M. M. 2010 Subcutaneous and visceral adipose tissue: structural and functional differences. Obes. Rev. 11:11–18. 19656312 10.1111/j.1467-789X.2009.00623.x

[phy212909-bib-0020] Kahn, S. E. 2003 The relative contributions of insulin resistance and beta‐cell dysfunction to the pathophysiology of Type 2 diabetes. Diabetologia 46:3–19. 12637977 10.1007/s00125-002-1009-0

[phy212909-bib-0021] Kakkar, A. K. , and N. Dahiya . 2015 Drug treatment of obesity: current status and future prospects. Eur. J. Intern. Med. 26:89–94. 25634851 10.1016/j.ejim.2015.01.005

[phy212909-bib-0022] Khan, L. K. , K. Sobush , D. Keener , K. Goodman , A. Lowry , J. Kakietek , et al. ; Centers for Disease Control and Prevention . 2009 Recommended community strategies and measurements to prevent obesity in the United States. MMWR Recomm. Rep. 58:1–26. 19629029

[phy212909-bib-0023] Konrad, D. , A. Rudich , and E. J. Schoenle . 2007 Improved glucose tolerance in mice receiving intraperitoneal transplantation of normal fat tissue. Diabetologia 50:833–839. 17334653 10.1007/s00125-007-0596-1

[phy212909-bib-0024] Kowalski, G. M. , and C. R. Bruce . 2014 The regulation of glucose metabolism: implications and considerations for the assessment of glucose homeostasis in rodents. Am. J. Physiol. Endocrinol. Metab. 307:E859–E871. 25205823 10.1152/ajpendo.00165.2014

[phy212909-bib-0025] Laforest, S. , J. Labrecque , A. Michaud , K. Cianflone , and A. Tchernof . 2015 Adipocyte size as a determinant of metabolic disease and adipose tissue dysfunction. Crit. Rev. Clin. Lab. Sci. 52:301–313. 26292076 10.3109/10408363.2015.1041582

[phy212909-bib-0026] Malik, V. S. , W. C. Willett , and F. B. Hu . 2013 Global obesity: trends, risk factors and policy implications. Nat. Rev. Endocrinol. 9:13–27. 23165161 10.1038/nrendo.2012.199

[phy212909-bib-0027] Ogden, C. L. , M. D. Carroll , B. K. Kit , and K. M. Flegal . 2014 Prevalence of childhood and adult obesity in the United States, 2011‐2012. JAMA 311:806–814. 24570244 10.1001/jama.2014.732 PMC4770258

[phy212909-bib-0028] Patel, P. , and N. Abate . 2013 Body fat distribution and insulin resistance. Nutrients 5:2019–2027. 23739143 10.3390/nu5062019 PMC3725490

[phy212909-bib-0029] Perrini, S. , A. Leonardini , L. Laviola , and F. Giorgino . 2008 Biological specificity of visceral adipose tissue and therapeutic intervention. Arch. Physiol. Biochem. 114:277–286. 18946788 10.1080/13813450802334752

[phy212909-bib-0030] Rasband, W. 1997 ImageJ Software, Java 1.6.0_33, version 1.44n9 ed.: National Institutes of Health. Available at http://imagej.nih.gov/ij/index.html. (accessed 30 July 2016).

[phy212909-bib-0031] Rosen, E. D. , and B. M. Spiegelman . 2006 Adipocytes as regulators of energy balance and glucose homeostasis. Nature 444:847–853. 17167472 10.1038/nature05483 PMC3212857

[phy212909-bib-0032] Rytka, J. M. , S. Wueest , E. J. Schoenle , and D. Konrad . 2011 The portal theory supported by venous drainage‐selective fat transplantation. Diabetes 60:56–63. 20956499 10.2337/db10-0697 PMC3012197

[phy212909-bib-0033] Satoor, S. N. , A. S. Puranik , S. Kumar , M. D. Williams , M. Ghale , A. Rahalkar , et al. 2011 Location, location, location: beneficial effects of autologous fat transplantation. Sci. Rep. 1:81. 22355600 10.1038/srep00081 PMC3216568

[phy212909-bib-0034] Seetharam, A. , V. Tiriveedhi , and T. Mohanakumar . 2010 Alloimmunity and autoimmunity in chronic rejection. Curr. Opin. Organ Transplant. 15:531–536. 20613527 10.1097/MOT.0b013e32833b31f4 PMC2926928

[phy212909-bib-0035] Tchernof, A. , and J. P. Despres . 2013 Pathophysiology of human visceral obesity: an update. Physiol. Rev. 93:359–404. 23303913 10.1152/physrev.00033.2011

[phy212909-bib-0036] Thomas, C. D. , J. C. Peters , G. W. Reed , N. N. Abumrad , M. Sun , and J. O. Hill . 1992 Nutrient balance and energy expenditure during ad libitum feeding of high‐fat and high‐carbohydrate diets in humans. Am. J. Clin. Nutr. 55:934–942. 1570800 10.1093/ajcn/55.5.934

[phy212909-bib-0037] Torres‐Villalobos, G. , N. Hamdan‐Perez , A. R. Tovar , G. Ordaz‐Nava , B. Martinez‐Benitez , I. Torre‐Villalvazo , et al. 2015 Combined high‐fat diet and sustained high sucrose consumption promotes NAFLD in a murine model. Ann. Hepatol. 14:540–546. 26019041

[phy212909-bib-0038] Tovar, A. R. , A. Diaz‐Villasenor , N. Cruz‐Salazar , G. Ordaz , O. Granados , B. Palacios‐Gonzalez , et al. 2011 Dietary type and amount of fat modulate lipid metabolism gene expression in liver and in adipose tissue in high‐fat diet‐fed rats. Arch. Med. Res. 42:540–553. 22023986 10.1016/j.arcmed.2011.10.004

[phy212909-bib-0039] Tran, T. T. , Y. Yamamoto , S. Gesta , and C. R. Kahn . 2008 Beneficial effects of subcutaneous fat transplantation on metabolism. Cell Metab. 7:410–420. 18460332 10.1016/j.cmet.2008.04.004 PMC3204870

[phy212909-bib-0040] Wong, R. H. , and H. S. Sul . 2010 Insulin signaling in fatty acid and fat synthesis: a transcriptional perspective. Curr. Opin. Pharmacol. 10:684–691. 20817607 10.1016/j.coph.2010.08.004 PMC3092640

